# Update and review of the current medical and surgical management of sickle cell retinopathy

**DOI:** 10.1186/s40662-026-00484-2

**Published:** 2026-04-27

**Authors:** Carson W. Ercanbrack, Sam Karimaghaei, Abdulrahman H. Badawi, Moustafa Magliah, Ahmed B. Sallam

**Affiliations:** 1 College of Medicine, University of Arkansas for Medical Sciences, Little Rock, AR USA; 2 Department of Ophthalmology, Harvey and Bernice Jones Eye Institute, University of Arkansas for Medical Sciences, Little Rock, AR USA; 3 Retina Division, King Khaled Eye Specialist Hospital, Riyadh, Saudi Arabia; 4 Department of Ophthalmology, Ain Shams University, Cairo, Egypt; 5 Department of Ophthalmology, Jones Eye Institute, University of Arkansas for Medical Sciences, 4301 W Markham Street # 523, Little Rock, AR 72205 USA

**Keywords:** Sickle cell, Sickle cell retinopathy, Proliferative sickle cell retinopathy, Non-proliferative sickle cell retinopathy, Sickle cell disease

## Abstract

**Background:**

Sickle cell retinopathy (SCR) is a well-documented and potentially vision-threatening presentation of sickle cell disease (SCD). In this article, we provide a comprehensive review of the management options for non-proliferative sickle cell retinopathy (NPSR) and proliferative sickle cell retinopathy (PSR) based on the existing ophthalmic literature.

**Main text:**

The mainstay of NPSR treatment focuses on preventing progression to PSR by identifying and altering modifiable risk factors. Once NPSR progresses to PSR, suppression of vascular endothelial growth factor (VEGF) expression with laser photocoagulation or intravitreal anti-VEGF injections can be considered. While no standard criteria exist for timing and type of intervention, both treatment modalities have been utilized for advanced PSR. In contemporary practice, scatter laser photocoagulation is performed far more commonly than the historically described feeder-vessel photocoagulation. Surgical management typically includes pars plana vitrectomy (PPV), scleral buckle (SB), or combined PPV-SB and are generally indicated in PSR for non-clearing vitreous hemorrhage, tractional or rhegmatogenous retinal detachment (RD), and epiretinal membrane formation.

**Conclusion:**

There is currently no consensus on standard guidelines for the management of SCR. Evidence suggests that surgical intervention can improve vision in advanced stages of PSR and that anti-VEGF therapy may have a role in treatment. However, studies in the ophthalmic literature are limited by relatively small sample sizes and difficulty accounting for a patient’s prior medical or surgical interventions. Additionally, more robust studies are required to determine the long-term efficacy and safety of anti-VEGF in SCR. A multidisciplinary team approach to SCR and SCD remains the cornerstone of management for this systemic disease.

## Background

There are currently no consensus guidelines for the treatment of sickle cell retinopathy (SCR) due to a paucity of robust randomized controlled clinical trials. In this article, we provide a comprehensive review of the management options for non-proliferative sickle cell retinopathy (NPSR) and proliferative sickle cell retinopathy (PSR) based on the existing ophthalmic literature. We performed a comprehensive review of the literature using the PubMed database using combinations of various keywords, including “management of,” “medical management of,” “surgical management of,” “sickle cell retinopathy,” “non-proliferative sickle cell retinopathy,” “proliferative sickle cell retinopathy.”

SCR is the most common ocular manifestation of sickle cell disease (SCD) [1]. About 0.5% of patients with homozygous sickle cell disease (HbSS) and 2.5% of patients with combined sickle cell and hemoglobin C disease (HbSC) will develop PSR [1]. SCD is a group of hemoglobinopathies caused by varying degrees of nucleotide mutations in the β-globin gene of hemoglobin A, which is the main type of hemoglobin in adult red blood cells (RBCs) and is comprised of two α-globin chains and two β-globin chains [2]. In HbSS, a point mutation causes the conversion of the codon cytosine-adenine-guanine (CAG) to cytosine-thymine-guanine (CTG) [3]. As a consequence, a negatively charged glutamine is replaced with a nonpolar valine on the sixth position of the β-globin chain [2]. This leads to the disease’s signature hemoglobin S. Unlike physiologically normal hemoglobin A, hemoglobin S polymerizes in hypoxic conditions and oxidative stress, causing RBC sickling. When sickled, the RBCs become more rigid, leading to a longer transit time across vessels and increased endothelial adhesions [1]. These combined factors lead to the release of inflammatory mediators, which promote further vascular occlusion [1].

Another hemoglobinopathy, hemoglobin C, is a common structural variation of hemoglobin A resulting from lysine substitution for glutamate in the sixth position of the β-globin chain [4]. This makes the mutated hemoglobin C less soluble than physiologically normal hemoglobin [4]. HbSC presents with a similar systemic disease to HbSS but is generally less severe [4]. While systemic symptoms are less common in HbSC than HbSS, retinopathy is more likely to occur in HbSC disease [4–6]. One theory for this is that HbSS is more likely to have auto-infarctions, leading to the regression of neoproliferative vessels [7]. Conversely, HbSC is more likely to have less severe and fewer overall occlusive events, leading to more hypoxic stress and consequential neovascularization [7]. Another theory for the increased prevalence of proliferative retinopathy in HbSC is that RBCs with hemoglobin C have high potassium-chloride cotransport activity, leading to potassium and water loss in the RBC [8]. The subsequent loss of potassium and water leads to greater sickling than would be expected with only one copy of the hemoglobin S gene.

SCR is mostly asymptomatic as the peripheral retina, especially the temporal part, is more commonly involved [1, 6]. In more advanced stages, patients may complain of flashes of light or floaters due to retinal traction or retinal detachment (RD). SCR can be categorized into two different classes: NPSR and PSR. PSR can also be further classified using a grading system created by Goldberg in 1971. The Goldberg stages are as follows: stage 1—peripheral arteriolar occlusions are present; stage 2—peripheral arteriolar-venular anastomosis is present; stage 3—neovascular and fibrous proliferation; stage 4—vitreous hemorrhage; stage 5—RD [9].

NPSR is the less severe form of SCR. Characteristic exam findings of NPSR include salmon patch hemorrhages, iridescent spots, and black sunburst lesions without the presence of neovascularization [10]. Salmon patch lesions are oval-shaped and indicative of superficial or pre-retinal hemorrhages [10]. Over time, these salmon patch hemorrhages are reabsorbed and degraded, leading to bright yellow spots at several layers of the retina [6]. These yellow spots are referred to as iridescent spots. Finally, hemorrhage in the outer retinal layers stimulates retinal pigment epithelium (RPE) proliferation, causing dark round or ovoid chorioretinal lesions termed black sunbursts [6]. Patients in the NPSR stages are generally asymptomatic. Red spots on the optic disc due to sickled RBCs (called the disc sign), posterior vessel tortuosity, angioid streaks, and abnormal red reflex due to macular ischemia (retinal depression sign) have also been reported as findings on dilated fundal exam of patients with NPSR [11]. In some cases, NPSR can also present as a central retinal artery occlusion (CRAO). As such, a characteristic cherry-red spot will be present in the macula.

NPSR may progress to PSR. As the names suggest, PSR is marked by the proliferation of retinal neovessels. Goldman stages 1–5 fall under PSR. The progression of NPSR to PSR results from recurrent hypoxic events that induce the release of vascular endothelial growth factor (VEGF), which drives subsequent neovascularization [12]. Unlike NSPR, PSR is a larger threat to vision and can be symptomatic if there is retinal traction, RD, or vitreous hemorrhage. Characteristic sea fans form at the borders of nonperfused zones in the peripheral retina [13]. Fibroglial proliferation over the sea fans results in fibrous bands that extend into the vitreous [6]. Sea fans may also auto-infarct, resulting in a white sea fan appearance [12]. Vitreous hemorrhages, corresponding to Goldman stage 4, usually result from bleeding of the newly formed vessels [13]. Tractional or combined tractional-rhegmatogenous RD, Goldman stage 5, are late complications of untreated and progressive fibrovascularization [13].

Numerous risk factors exist for the progression of NPSR to PSR. In a study of 953 patients with SCR, there was a statistically significant increase in the progression to PSR among patients with a history of smoking, increased hemoglobin level, white blood cell count, and older age [7]. In the same study, previous blood transfusion, high reticulocyte count, and high hemoglobin F were identified as protective factors against the progression to PSR [7].

Currently, no standard, agreed-upon screening guidelines exist. However, one editorial review did recommend annual ophthalmologic screening starting at age 10 in patients with SCD with ultra-widefield fundus photography (UWF-FP) and optical coherence tomography (OCT) [14]. Similarly, the National Heart, Lung, and Blood Institute recommends that all patients who are 10 years old or older with SCD be screened for retinopathy with dilated funduscopic exams every 1–2 years [15]. However, this recommendation is also based on expert consensus with relatively low-quality evidence [16]. One study reports that patients with previous pain crises or splenic sequestration, markers of HbSS progression, should consider having earlier ophthalmologic evaluation [17]. Additionally, the development of any new visual symptoms warrants referral to ophthalmology [7]. Nevertheless, due to the lack of guidance on best screening practices, there is substantial variability in practice [16]. A standardized practice regarding ideal screening protocol remains a significant gap in the management of SCR.

Optical coherence tomography angiography (OCTA) has also proven beneficial for screening for avascular zones and early macular ischemia [11]. One case series comprising 11 eyes from 10 patients with HbSS and one with HbSC used OCTA in conjunction with adaptive optics scanning light ophthalmoscopy (AOSLO) [18]. In the case series, investigators first obtained an OCTA centered around the fovea. Additional OCTA scans of the same eye were used to identify parafoveal capillaries with poor perfusion. Once these regions were identified, AOSLO imaging of the area of interest was acquired. Investigators were then able to directly visualize intermittent blood cell flow, blood cell stasis, blood cell sickling, and sites of thrombus formation. The authors proposed that combined OCTA–AOSLO imaging could be used to stage SCR, monitor disease activity, and assess response to systemic therapy [18]. However, this study had notable limitations. AOSLO provides a limited field of view, restricting its utility for widefield assessment [18]. Additionally, the small sample size precluded differentiation of AOSLO findings among NPSR, PSR, and between HbSS and HbSC genotypes [18]. Despite these limitations, combined OCTA–AOSLO imaging may represent a valuable tool for future SCR research.

## Main text

### Management of NPSR

The mainstay of NPSR treatment focuses on preventing progression to PSR. Currently, NPSR has no specific preventive or medical management. Instead, NPSR is managed by identifying and altering modifiable risk factors.

Amongst these modifiable risk factors, a history of smoking appears to be the most modifiable. One study, consisting of 953 participants, conducted by Nawaiseh et al. [7] reported that patients with a history of smoking have a statistically significant chance (*P* = 0.005) of progression to PSR. This correlates to findings found in other studies that demonstrate patients with HbSS who have a history of smoking are at increased risk for complications related to sickle cell anemia, such as pain crises, acute chest syndrome, pulmonary function abnormalities, and death [19–22]. An abstract by Khan et al. [23] reported that a history of cigarette smoking was associated with an increased risk of CRAO in patients with HbSS than in patients with HbSS who did not have exposure to cigarette smoke. Compounding these risks with the risks associated with cigarette smoking, smoking cessation counseling should be offered at every visit.

When examining systemic manifestations of SCD, vaso-occlusive episodes and their sequelae were significantly associated with a risk of SCR and PSR. Patients with aseptic necrosis, hematuria, leg ulcers, painful crisis, and hand-foot syndrome had a significantly increased risk of PSR [7]. This may be because these conditions are associated with a greater burden of uncontrolled disease. This underscores the need for systematic management to reduce the risk of SCR progression.

Hydroxyurea, also called hydroxycarbamide, has long been the mainstay of treatment for HbSS. By inducing fetal hemoglobin (HbF) production, hydroxyurea decreases the amount of pathologic RBCs that can sickle, ultimately decreasing the severity of sickling in hypoxic conditions [24]. Many studies suggest that high levels of HbF play a protective role in the prevention of PSR [7, 14, 25–27]. A study by Mian et al. [26] identified that HbF levels should be above 15% to exert retinal protective effects, reducing the odds of developing retinopathy by 50% in patients above the 15% threshold. Estepp et al. [25] found that children with HbSS and a HbF less than 15% have a 7.1-fold higher odds of developing retinopathy. Therefore, ensuring patients with HbSS are being managed appropriately with hydroxyurea by their primary hematologist is warranted.

The previously mentioned study by Nawaiseh et al. [7] consisting of 953 patients also found that previous blood transfusions were associated with protection against PSR. Like hydroxyurea, therapeutic transfusions can dilute the amount of sickled RBCs. However, caution should be taken when using transfusions as a means of treatment due to the risk of iron overload and hyperviscosity [7]. Further studies need to be conducted before definitive conclusions can be made regarding the protective effect of therapeutic transfusions against the development of PSR.

Many non-modifiable risk factors exist as well, including HbSC, male sex, and older age [5, 7, 14]. The correlation between PSR and male sex is unclear and may be confounded by the increased incidence of smoking in men compared to women [28]. Duan et al. [29] propose that males may be more prone to PSR due to their higher levels of hemoglobin compared to females. Some studies hypothesize that estrogen can protect against endothelial dysfunction by alteration of nitric oxide [30–32].

### Laser therapy for PSR

Specific interventions in Goldberg stages 1 and 2 are not indicated, as treating the ischemic retina does not prevent the formation of sea fan retinal neovascularization [6]. Treatments are generally considered when retinopathy reaches the proliferative stages, Goldberg stage 3 and beyond. No distinct set of parameters exists in determining when to initiate treatment in Goldberg stage 3, but most retina specialists will offer treatment if there is bilateral PSR, large elevated sea fans with active neovascularization, associated vitreous hemorrhage, and vision loss in the fellow eye [33, 34]. A study in 2022 by Griffin et al. [35] found that among 97 cases of scatter laser photocoagulation for 55 patients with PSR, 68.4% were done due to retinal capillary non-perfusion and retinal neovascularization, 21.5% for vitreous hemorrhage, and 10.1% for tractional RD. However, the benefit of laser treatment is unclear. Fox et al. [36] found that among 88 patients with HbSC treated with scatter laser therapy, only patients less than 25 years old demonstrated a regression of neovascularization. Nagpal et al. [37] noted spontaneous sea fan regression in 45 patients without any intervention but still recommended treating proliferative disease as opposed to observation [37].

Two main forms of laser therapy exist for PSR: feeder vessel coagulation and scatter laser photocoagulation. Feeder vessel coagulation involves direct closure of neovessels by applying heavy laser burns to the arterioles supplying them [34]. This can be done with argon laser or historically with xenon arc, both of which have been shown to reduce the incidence of vitreous hemorrhage and subsequent vision loss [38]. Argon laser is generally preferred as there is a lower associated risk of choroidal neovascularization as compared to the use of xenon arc [38]. Xenon arc also tends to cause more visual field damage and defects on electroretinogram compared to argon laser [39]. However, argon laser feeder vessel coagulation carries a higher risk of iatrogenic rhegmatogenous RD [38, 40]. With the unfavorable side effect profile of feeder vessel coagulation, it is no longer carried out in modern-day practice, and scatter laser photocoagulation is the main stay of treatment. Scatter laser photocoagulation has an indirect effect on PSR by reducing the release of VEGF from an ischemic retina [33].

In the first randomized clinical trial of scatter laser photocoagulation for PSR, Farber et al. [40] found that eyes treated with laser demonstrated significantly lower rates of visual acuity (VA) loss and vitreous hemorrhage [40]. The burns used had a spot size of 500 μm and a duration of 0.1 s. Burns were placed approximately one burn diameter apart and were usually placed from one disc diameter posterior to one disc diameter anterior to the sea fan and one clock hour to each of the sides. Fox et al. conducted a study consisting of 74 patients with HbSC treated with scatter laser photocoagulation and compared to 60 patients with HbSC that served as a control group [36]. Burns were 500 μm in diameter, 0.1 s duration, and placed around the PSR lesions. The number of burns depended on the size of the PSR lesion. The authors found that rates of PSR regression on fluorescein angiography were statistically higher in treated eyes only in patients younger than 25 years (*P* < 0.001) and that there was no significant difference among treated and untreated patients older than 25 years (*P* = 0.6) [36]. Lesions in this study were also more likely to infarct in treated eyes if the lesions were small, and if the lesions were flat [36]. In the median follow-up period of 2.9 years, 34% of treated eyes and 58% of untreated eyes developed new PSR lesions after laser treatment [36]. In both studies, there were no reported complications attributed to the photocoagulation therapy.

Sayag et al. [41] further divided Goldberg stage 3 into stages A, B, C, D, and E depending on the size of the sea fan, presence of hemorrhage, fibrosis, and visibility of vessels. The proposed grading system is as follows: grade A is a flat sea fan with leakage of less than one macular photocoagulation study disc area, grade B is an elevated sea fan with hemorrhage, grade C is an elevated sea fan with partial fibrosis, grade D complete sea fan fibrosis without well-demarcated vessels, and grade E complete sea fan fibrosis with well-demarcated vessels [41]. In their study of 38 patients receiving scatter laser photocoagulation and 35 receiving no treatment, Sayag et al. found that the progression of PSR between treated and untreated eyes was not statistically significant in grades A and C. However, there was a statistically significant decrease in progression and increased regression of PSR in grade B eyes that received laser treatment. Additionally, there was a statistically increased number of complications that occurred in untreated eyes in grades B and E. Nine complications were reported: three retinal tears and six vitreous hemorrhages [34, 41]. All eyes within grade D did not demonstrate progression, regression, or complications within the mean follow-up period of 4 years [41].

In a recent 2022 single-center retrospective study [42] of 55 eyes in Goldberg stage 3 treated with laser photocoagulation, VA after treatment was unchanged in 61.8% of eyes, improved in 20%, and worse in 18.2% [42]. Sea fan vessels regression occurred in 90.9% of eyes after laser, and no adverse effects were reported [42]. In this study, the treatment protocol extended one millimeter anterior and posterior with one clock hour on each side of the sea fan. The number and extent of burns depended on lesion size and the surgeon's discretion.

### Cryotherapy for PSR

There remains a scarcity of data comparing the efficacy of laser photocoagulation and cryotherapy in preventing RD. Existing studies primarily evaluated these modalities, along with scleral buckling, after RD has occurred. Although VA between the two groups was not statistically significant at 10 weeks, the cryopexy group had a significantly higher postoperative flare and a slower recovery than the laser photocoagulation group [43]. Nonetheless, cryopexy does have some advantages. Unlike laser photocoagulation, cryopexy avoids tissue vaporization and can be used when visualization is limited by media opacification [44]. Additionally, when the retina is elevated, laser uptake may be blunted from subretinal fluid, making cryotherapy a preferable option.

Evidence supporting the use of cryotherapy in PSR is also limited. A 1971 study reported complete closure of 28 sea fans in nine patients with three cycles of cryopexy [45]. However, in this study, two of the nine eyes subsequently developed RD. Based on these outcomes, the authors advised against repeated cryopexy, suggesting that tractional force on the retina may exacerbate the risk of detachment with multiple cryotherapy cycles. In a separate 1982 study, scatter cryotherapy and photocoagulation successfully obliterated peripheral neovascularization in seven eyes of five patients [46]. More contemporary and robust studies are needed to evaluate the effectiveness of cryopexy in modern management of PSR.

### Intravitreal anti-VEGF factor for PSR

The success of intravitreal anti-VEGF agents in the treatment of proliferative diabetic retinopathy (PDR) and wet age-related macular degeneration has prompted consideration of their use in PSR [47, 48]. Compared to normal eyes, VEGF expression is increased in eyes with untreated PSR [49]. However, long-term studies evaluating their efficacy in PSR remain scarce, with various case reports and case series showing mixed results.

Among 70 eyes with PSR receiving anti-VEGF injections in a single-center retrospective study by Obeng et al. [50], VA improved in all eyes. The 30 eyes without prior intervention (no laser photocoagulation scars) did not develop any complications in the mean follow-up period of 6 ± 1 years. Contrarily, all 40 eyes that had prior laser photocoagulation developed recurrent proliferative vessels, which were subsequently treated with anti-VEGF injections. On average, at least three anti-VEGF injections were needed to control PSR without detachment. Of note, this study mixed aflibercept, bevacizumab, and ranibizumab due to resource scarcity, and no single anti-VEGF agent was able to be denoted as superior.

In their retrospective interventional study of 108 eyes with PSR, Okonkwo et al. [51] identified five treatment groups: (1) intravitreal anti-VEGF monotherapy, (2) scatter laser photocoagulation only, (3) pars plana vitrectomy (PPV) and scatter laser photocoagulation, (4) Intravitreal anti-VEGF and scatter laser photocoagulation, (5) PPV, intravitreal anti-VEGF, and scatter laser photocoagulation. Intravitreal anti-VEGF was primarily used in Goldberg stage 4 PSR (81.4% of eyes). Scatter laser photocoagulation was used mainly to treat Goldberg stage 3 (42.9% of eyes) and stage 5 (57.1% of eyes). Intravitreal anti-VEGF and scatter laser photocoagulation-treated eyes had the best pre- and post-treatment VA. Eyes that underwent vitrectomy and scatter laser photocoagulation had the most improved vision. Scatter laser photocoagulation only was associated with the least improvement. Overall, 90.7% of eyes had improved or maintained VA post-treatment.

A study by Lim et al. [52] investigated intravitreal anti-VEGF for Goldberg stages 3 and 4 PSR. Thirty-three eyes total were examined with retrospective chart review, 15 of which were in stage 3, and 18 in stage 4. Ten eyes had prior laser treatment. Thirteen eyes were given anti-VEGF alone and 20 eyes anti-VEGF followed by laser photocoagulation. Nine eyes treated with anti-VEGF alone had sea fan regression at 1 month. Among eyes with more than 1-month follow-up, 87.5% of those treated with anti-VEGF alone and 85% treated with both anti-VEGF and photocoagulation had inactive sea fans. Furthermore, 12 of 18 eyes with stage 4 PSR and baseline VA of 20/20 or 20/25 had improvement in VA greater than three lines, 5 of 18 had stable VA, and only one eye had worsened VA at 1-month follow-up after anti-VEGF injection. In 12 of 13 eyes with stage 4 PSR and baseline VA 20/40 or worse, VA improved by greater than three lines at 1-month follow-up after anti-VEGF injection. Thus, investigators concluded that anti-VEGF therapy resulted in a high rate of sea fan regression and stable or improved VA in the majority of eyes.

Another vital consideration regarding anti-VEGF therapy is that it should not be used as monotherapy when fibrovascular proliferation is present [53, 54]. Although these studies examined anti-VEGF use in the setting of PDR, they demonstrate the potential for accelerated fibrosis after intravitreal anti-VEGF therapy and subsequent tractional RD. Anti-VEGF agents, conversely, have demonstrated utility as preoperative adjuncts in PSR [47]. While the presented studies demonstrate a promising role for anti-VEGF use in PSR, studies to determine the long-term efficacy and safety of these injections must be conducted before they are standardized.

### Other anti-angiogenic mediators for PSR and their potentials

Like VEGF, angiopoietin-1 and -2 are additional cytokines implicated in retinal neovascularization [55–57]. Angiopoietin-1 supports the maintenance and stabilization of mature vessels, whereas angiopoietin-2 promotes vessel destabilization [55]. Angiopoietin-2 is also upregulated in hypoxic conditions and by angiogenic cytokines, such as VEGF [58, 59]. Because of this, Mohan et al. [55] investigated the use of plasma levels of angiopoietin-1 and angiopoietin-2 in patients with SCR, both NPSR and PSR. Additionally, they measured these cytokines after PRP. The investigators found that patients with SCR had higher plasma angiopoietin-1 and -2 levels than control patients, and that angiopoietin-1 increased significantly following PRP. The authors also propose using an “angiogenic index,” defined as the angiotensin-2-to-VEGF ratio, as a potential indicator of PSR development. A low index would indicate a tendency to neovascularization, whereas a low index would indicate a propensity to neovascular regression. However, this study did not demonstrate any angiopoietin or VEGF pattern associated with retinopathy severity.

The role of angiopoietin as a marker of disease burden and progression is further supported by an additional study by Andrawes et al. [56], in which angiopoietin-2 levels were significantly higher in patients with HbSS than in controls. Additionally, an angiopoietin-2 level of 9000 pg/mL or higher was able to detect retinopathy with 100% sensitivity and specificity.

These studies are promising and may support the potential use of serum angiopoietin as a screening tool for SCR. A relationship between disease burden and angiopoietin-2 is further strengthened by an additional study that demonstrated a significant decrease in levels after initiation of hydroxyurea [57]. Nonetheless, further studies remain warranted. The studies presented have relatively small sample sizes, and larger cohorts are required to establish reliable threshold values and further clarify the clinical utility of angiopoietin-2 in SCR. Given the role of angiopoetin-2 in SCR, future investigations may also explore the efficacy and safety of intravitreal faricimab, which blocks both VEGF and angiopoetin-2 [60].

Similarly, hypoxia-inducible factor-1α (HIF-1α) is another angiogenic mediator involved in SCR [66]. In a 2016 study by Rodrigues et al. [49], five eyes from patients with known PSR were autopsied and compared to three control eyes. The investigators found that HIF-1α and VEGF were expressed in neovascular sea fans and within the inner retina in the area between the perfused and nonperfused retina. The control eyes showed no detectable HIF-1α expression in the inner retina. This study is limited because only autopsied eyes were examined and tested. Additionally, the sample size was small. If future research can confirm these findings, it may lead to additional therapeutic options.

### Surgical management for PSR

**Fig. 1:**
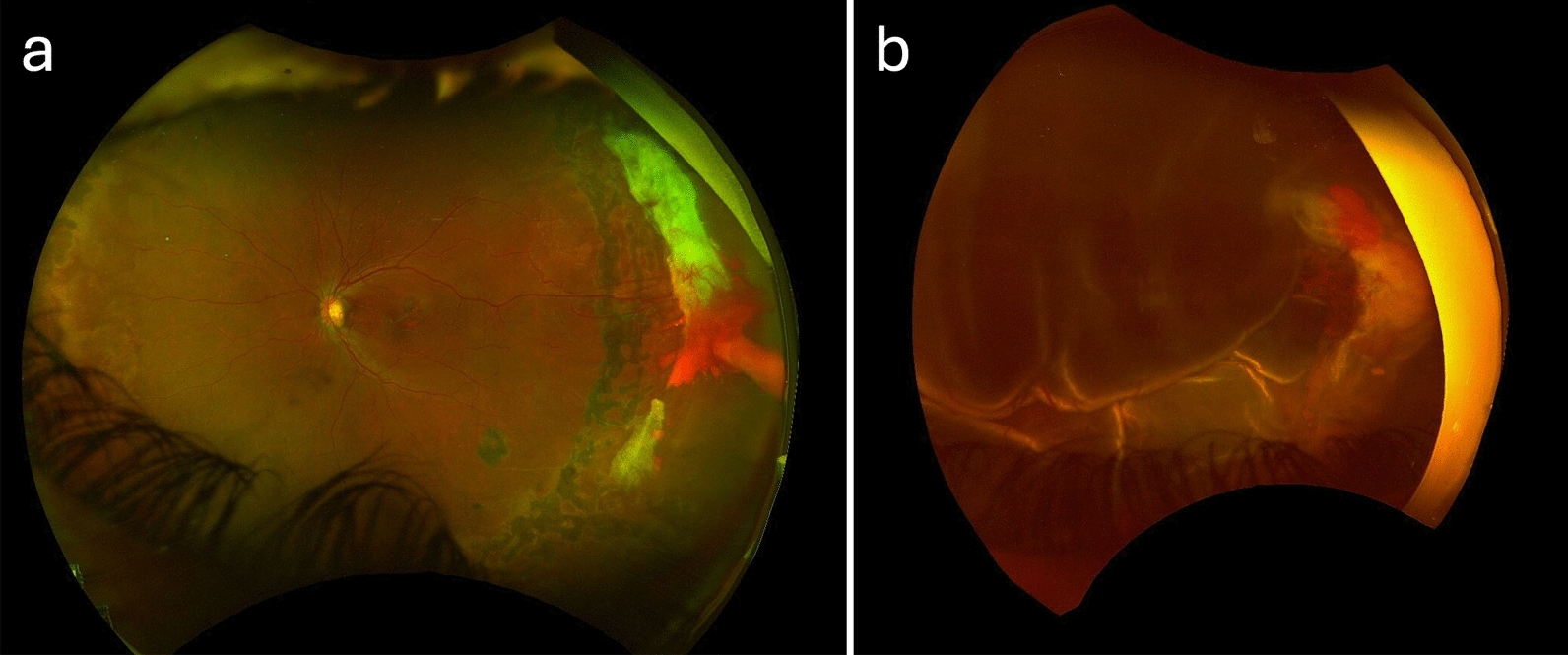
Pseudo-color fundus photographs of a left eye with proliferative sickle cell retinopathy (PSR) taken 2 years apart. **a** Demonstrates peripheral laser photocoagulation scars and extensive peripheral ischemia with temporal foci of fibrotic regressed sea fans and an active sea fan with overlying vitreous hemorrhage. **b** The same eye 2 years later with severe tractional rhegmatogenous retinal detachment (RD) and vitreous hemorrhage

Surgical management for PSR is generally indicated when more severe retinal complications occur, most commonly non-clearing vitreous hemorrhage, traction RD, rhegmatogenous RD, and epiretinal membrane formation [6, 61, 62]. Figure 1 demonstrates a case of PSR progressing to combined tractional-rhegmatogenous RD requiring surgical intervention. The patient underwent combined phacoemulsification cataract extraction and PPV, during which a large macular hole was identified and silicone oil tamponade used. They subsequently required reoperation with silicone oil tamponade due to persistent RD and thick epiretinal membrane formation. The postoperative course was complicated by pupillary block with persistent ocular hypertension after laser peripheral iridotomy due to high peripheral anterior synechiae and required Ahmed tube shunt implantation with pupillary membrane removal. The patient continued to have persistent subretinal fluid after reoperation, but further surgical intervention was deferred due to poor visual prognosis. These are original photographs obtained by manuscript authors.

To prevent vaso-occlusion and ischemia, careful preoperative planning should take place [62]. Freilich and Seelenfreund [63] performed scleral buckling in three patients with PSR using a hyperbaric oxygen chamber without any adverse effects, suggesting that combining scleral buckling with hyperbaric oxygen therapy may prevent anterior segment ischemia (ASI). However, few studies have evaluated the necessity of hyperbaric oxygen therapy beyond a few case reports [64, 65].

Preoperative exchange transfusion has also been attempted to prevent ASI [66]. However, Pulido et al*.* [67] proposed that it is unnecessary if adequate hydration, oxygenation, and intraocular pressure are maintained preoperatively by a multidisciplinary team with a hematologist. In their study, five out of 11 patients with PSR received preoperative exchange transfusion. Neither transfused nor non-transfused patients developed postoperative ASI [67].

A review of perioperative management for PSR by Nithianandan and Sridhar [62] found that most studies avoided retrobulbar anesthesia and the use of epinephrine to prevent vasoconstriction and ischemic complications. They also emphasized that the need for preoperative transfusions was not well established in ophthalmic literature. Finally, while hydroxyurea is recommended to patients with SCD for a variety of reasons, the perioperative role of the medication has yet to be studied [62].

Outside of ophthalmic literature, the Transfusion Alternatives Preoperatively in Sickle Cell Disease Study found that patients undergoing intrabdominal and orthopedic surgery have fewer complications, including acute chest syndrome [68]. In such surgeries, some studies recommend a hemoglobin level of 10 g/dL or higher [69]. Standard supportive measures such as adequate intravenous and oral hydration to avoid dehydration-induced sickling [70] and ensuring proper intraoperative oxygenation [71] are also essential components of safe perioperative care.

Despite these varied preoperative and perioperative considerations, surgical outcomes for advanced PSR can be favorable. Oderinlo et al. [72] found that among 22 eyes with Goldberg stages 4 or 5 PSR, all eyes achieved anatomic success at postoperative week 1, 6, and 12 regardless of surgical procedure performed: PPV, combined PPV and a scleral buckle (SB), or combined PPV-phacoemulsification cataract surgery. There was also a statistically significant improvement in mean postoperative VA.

As with other retinal surgeries, preoperative imaging is crucial for surgical planning and to minimize complications. In SCR, visualization of the peripheral fundus is particularly important for characterizing disease burden and identifying subtle peripheral vascular changes or ischemia that may not be detected by clinical examination alone. The role of imaging is further highlighted when vitreous hemorrhage, cataract, or another media opacity precludes direct fundus examination. Imaging that has proven helpful in the setting of PSR includes UWF-FP and ultra-widefield fluorescein angiography [11, 12]. As previously discussed in this manuscript, OCTA has also proven beneficial for evaluating for avascular zones and early macular ischemia. Unfortunately, conventional OCTA is limited by its small field of view, which restricts simultaneous visualization of the posterior pole and peripheral retina [73]. While widefield OCTA has become increasingly accessible in clinical practice, it remains in its early stages and continues to require ongoing technical improvement [73]. Widefield OCTA remains an essential unmet need for SCR.

### Scleral buckle

Before the introduction of modern vitrectomy surgery, PSR that required surgical intervention was managed with a circumferential SB [62, 66]. However, patients with PSR who underwent scleral buckling were found to have a higher incidence of anterior segment ischemia (ASI) due to possible sickling within the anterior ciliary arteries, damage to the long posterior ciliary arteries by the placement of the buckle itself, or any combination of these factors [74]. With the concern for ASI and the available option of PPV, using circumferential scleral buckling alone for RD in the setting of PSR has largely fallen out of favor and, as such, ASI related to posterior segment surgery for PSR has decreased [62]. Nithianandan and Sridhar [62] also pointed out that circumferential SBs today if to be used in PSR-related RD are not typically placed as high and broad as they were in the past, which may also be a contributing factor to the lowered incidence of ASI. Even with advances that have reduced ASI rates in circumferential scleral buckling, the procedure by itself is still less commonly preferred relative to PPV that offers superior alleviation of vitreoretinal traction compared with scleral buckling alone [61, 72, 75].

Segmental scleral buckling is a variant of traditional SB that provides local support to specific areas of the retina where RD or breaks may be present [76]. Unlike circumferential buckles, segmental SBs in rhegmatogenous RD have decreased postoperative complications such as myopia, extraocular muscle imbalance, and scleral erosions [76] without compromising on anatomical success [77]. However, to our knowledge, no studies have evaluated the effectiveness and safety of segmental SBs in patients with SCR related RD.

### Pars plana vitrectomy

PPV was first reported as a means for PSR management in 1988 by Pulido et al. [67]. In this 11-patient study, no patient experienced ASI. In another retrospective interventional case series consisting of 28 eyes in Goldberg stages 4 and 5 compared 10 eyes that underwent observation to 18 eyes that underwent PPV [78]. Two of the 10 eyes that were observed, one with rhegmatogenous RD and the other with tractional RD with epiretinal membrane, had spontaneous flattening of the retina. Of the 18 eyes that received PPV, 11 had postoperative complications, which included seven iatrogenic breaks, two postoperative vitreous hemorrhages, and three cataracts. Despite the complications, 15 of 18 eyes (83%) that underwent PPV had statistically significant improvement in vision postoperatively (*P* = 0.001), which is higher than the 50% reported by Cohen et al. [79] in 1986. This difference may be attributed to modern-day advancements in PPV.

Williamson et al. [78] reported a retrospective case series that included18 patients with vitreoretinal complications from PSR requiring PPV. The rate of iatrogenic retinal tears in this series was approximately 40%. The authors suggested that the high rate of iatrogenic tears in PPV surgery for PSR is a consequence of the technical difficulty of delaminating sea fan complexes and propose that segmentation without delamination is a more favorable approach to relieve retinal traction. The patients in this study did not undergo any additional scleral buckling.

Ho et al. [80] found success using 23-gauge vitrectomy compared to 20-gauge vitrectomy for PSR-associated RDs, tractional vitreous hemorrhages, and macular holes [80]. Both 23-gauge and 20-gauge vitrectomies had statistically significant improvements in postoperative VA. Of the two, 23-gauge yielded a slightly improved Early Treatment Diabetic Retinopathy Study (EDTRS) chart of 32 versus the 25 EDTRS of the 20-gauge cohort. However, there was no statistically significant difference between the two. There were also fewer, albeit not statistically significant, perioperative complications of four (three entry site breaks and one fibrinous uveitis) when using the 23-gauge compared to the seven (three entry site breaks, two giant retinal tears, two branch retinal artery occlusions, and one hypertensive uveitis) when a 20-gauge was used. While the results did not reveal statistical significance, using a 23-gauge vitrectomy demonstrated promising trends as a 23-gauge is more efficient and faster, and lends itself to less trauma and risk intraoperatively [80].

### Pars plana vitrectomy with scleral buckle

In a retrospective consecutive case series by Rohowetz et al. [75] of 65 eyes, 52 eyes with PSR-associated RD underwent PPV or combined PPV with a SB. Compared to the 23 eyes that underwent PPV alone, the 29 eyes that had combined PPV and SB had a higher rate of single-operation success. While the rates of single operation success with a combined procedure were higher (72.4%) than that of PPV alone (47.8%) the differences were not statistically significant (*P* = 0.07). The two groups also did not have a statistically different postoperative VA (*P* = 0.48). Another case series by Chen et al. [61] revealed that 4 of 8 patients who underwent initial PPV for PSR-associated tractional and rhegmatogenous RD had a recurrence of RD and required another surgery. Despite the use of SBs in these two studies, no ASI was reported [61, 72, 75]. Therefore, with advancements to the PPV procedure alongside the avoidance of high broad buckles, PPV in conjunction with SB may be a feasible option of reducing the need for a second intervention in cases of PSR-associated RD [61, 72].

### Sickle cell maculopathy

Although the primary focus of this review is the management of SCR, it is crucial to acknowledge sickle cell maculopathy (SCM) as a distinct and clinically significant ocular manifestation within the spectrum of SCD. SCM is currently defined as patchy areas of retinal thinning in the perifoveal temporal macula or within the macula itself [81]. While not always clinically evident on ophthalmoscopy, SCM is more readily identified on OCTA and spectral-domain optical coherence tomography (SD-OCT) [82]. With the temporal macular thinning visualized on SD-OCT, OCTA can identify signs of macular ischemia, such as irregularities of the foveal avascular zone, extension of no-flow areas, and decreased general vascular density [81]. The etiology of macular thinning remains poorly elucidated; a few studies suggest a primary vascular dysfunction or local macular ischemia [83, 84]. Many studies report a higher prevalence of SCM in patients with PSR, indicating a potential relationship between macular and peripheral ischemia [82, 85, 86].

Additionally, some studies report a higher frequency of SCM in patients with HbSS than in those with HbSC [81, 84, 87, 88]. However, this relationship remains inconsistent, as some studies reported a higher frequency of SCM in patients with HbSC [27, 89, 90]. While Dell’Arti et al. reported that the odds of severe retinal thinning decreased by 12.9% with a 1% increase in HbF, other studies have not identified a statistically significant association between HbF levels and SCM [82]. Thus, further interventional studies are needed to confirm the relationship between hydroxyurea, HbF levels, and SCM.

Cases of acute symptomatic macular ischemia in patients with HbSS have also been reported [91, 92]. In a case series of two patients with HbSS who presented with visual distortions and were found to have arterial occlusion of the posterior pole [91]. One patient received three exchange transfusions, and the other received one blood transfusion on presentation to the emergency department. Only the one patient who received exchange therapy reported subjective visual improvement at 1-month follow-up. Therefore, the investigators posit that exchange transfusions may be beneficial in cases of acute macular ischemia as they reduce the number of pathologic RBCs. However, managing macular ischemia requires ongoing research to establish the role of exchange transfusions in a larger patient group, as well as the effectiveness of hydroxyurea and other interventions that may reduce macular ischemia.

### Diabetes mellitus and SCD

Diabetes mellitus (DM), encompassing both type 1 (T1DM) and type 2 (T2DM), is a well-established risk factor for ocular complications, particularly diabetic retinopathy (DR) [93]. There is increasing evidence and growing consensus that HbSS and sickle cell trait (SCT) may hasten the progression of DR and other microvascular complications associated with DM [94–96]. A study by Skinner et al. [97] identified factors such as increased arterial stiffness, elevated blood viscosity, and higher concentrations of plasma advanced glycation end-products in patients with SCT and T2DM. These findings support the theory that the combined effects of vascular damage, inflammation, and oxidative stress, which are common in both HbSS, SCT, and DM, may contribute to the faster progression of DR when a patient has both diseases. Additionally, other cases have reported a synergistic effect between HbSS or SCT and DM on the development of retinopathy [98–100].

A large-scale population-based cohort study by Chauhan et al. [94] compared the risk of DR among patients with DM with and without HbSS or SCT. The study revealed a statistically significant increased risk of both non-proliferative diabetic retinopathy (NPDR) and PDR in patients with T2DM and HbSS compared to patients with T2DM alone. Moreover, patients with SCT had a statistically significant higher incidence of PDR in the presence of either T1DM or T2DM compared with those with T1DM or T2DM alone. These results underscore the importance of considering DM, particularly T2DM, in the screening, prognosis, and management of patients with coexisting sickle cell disorders. Additionally, this study highlights the importance of optimal DM control in this patient population to mitigate the progression of retinopathy.

A retrospective case series by Rohowetz et al. [101] evaluated the surgical outcomes in 20 eyes with SCR with concomitant DR. PPV was performed in all eyes, with one eye receiving a SB at the time of PPV. Indications for surgery were tractional RDs (12 eyes), combined tractional and rhegmatogenous RDs (six eyes), and vitreous hemorrhage (two eyes). This case series found no significant difference in pre- and postoperative best-corrected visual acuity (BCVA) after a mean follow-up of 40 months. Mean preoperative BCVA was 1.6 logarithm of the minimal angle of resolution (logMAR), while mean postoperative BCVAwas 1.5 logMAR. Among the 17 eyes that were treated for RD, only 11 achieved anatomic success at 6 months postoperatively, and 4 of the 11 eyes with anatomic success had retained silicone oil tamponade. These findings show relatively poor functional and anatomic outcomes in patients with SCR and DR who undergo surgical intervention. As such, patients should be counseled appropriately preoperatively [101]. This case series also suggests the need for surveillance in such patients to decrease the need for surgical intervention.

## Conclusion

Management of SCR proves to be complex, as no true standard of treatment exists and can differ by the treating ophthalmologist’s preferences and experiences. Studies in ophthalmic literature are limited by their retrospective design, relatively small sample sizes, and difficulty accounting for a patient’s prior medical or surgical interventions. Nonetheless, the studies mentioned earlier demonstrate a promising role for surgical intervention to improve VA in advanced stages of PSR. Additionally, depending on the long-term efficacy and safety, intravitreal anti-VEGF injections in SCR may expand on additional treatment modalities or as an adjunct treatment to reduce progression to higher stages of SCR. Randomized clinical trials are needed to allow for standardization of the management of PSR. A multidisciplinary team approach, comprising a hematologist or other SCD specialist and an ophthalmologist, remains vital for the treatment and management of this complex disease entity.

## Data Availability

Not applicable.

## Abbreviations

AOSLO: Adaptive optics scanning light ophthalmoscopy
ASI: Anterior segment ischemia
BCVA: Best-corrected visual acuity
CAG: Cytosine-adenine-guanine
CRAO: Central retinal artery occlusion
CTG: Cytosine-thymine-guanine
DM: Diabetes mellitus
DR: Diabetic retinopathy
ETDRS: Early Treatment Diabetic Retinopathy Study
HbF: Fetal hemoglobin
HbSC: Combined sickle cell and hemoglobin C disease
HbSS: Homozygous sickle cell disease
HIF-1α: Hypoxia-inducible factor-1α
LogMAR: Logarithm of the minimal angle of resolution
NPDR: Non-proliferative diabetic retinopathy
NSPR: Non-proliferative sickle cell retinopathy
OCT: Optical coherence tomography
OCTA: Optical coherence tomography angiography
PDR: Proliferative diabetic retinopathy
PPV: Pars plana vitrectomy
PPV-SB: Combined pars plana vitrectomy and scleral buckle
PSR: Proliferative sickle cell retinopathy
RBC: Red blood cell
RD: Retinal detachment
RPE: Retinal pigment epithelium
SB: Scleral buckle
SCD: Sickle cell disease
SCM: Sickle cell maculopathy
SCR: Sickle cell retinopathy
SCT: Sickle cell trait
SD-OCT: Spectral-domain optical coherence tomography
T1DM: Diabetes mellitus type 1
T2DM: Diabetes mellitus type 2
UWF-FP: Ultra-widefield fundus photography
VA: Visual acuity
VEGF: Vascular endothelial growth factor
